# A Critical Period for the Rapid Modification of Synaptic Properties at the VPm Relay Synapse

**DOI:** 10.3389/fnmol.2017.00238

**Published:** 2017-07-25

**Authors:** Libiao Pan, Junhua Yang, Qian Yang, Xiaomeng Wang, Liya Zhu, Yali Liu, Huifang Lou, Chou Xu, Ying Shen, Hao Wang

**Affiliations:** ^1^Department of Neurobiology, Key Laboratory of Medical Neurobiology of Ministry of Health of China, Key Laboratory of Neurobiology, Zhejiang University School of Medicine Hangzhou, China; ^2^Nanlou Respiratory Diseases Department, Chinese PLA General Hospital Beijing, China; ^3^Second Affiliated Hospital, Zhejiang University School of Medicine Hangzhou, China

**Keywords:** synaptic plasticity, critical period, somatosensory system, thalamus, development

## Abstract

In addition to cortical areas, the thalamus also displays plasticity during a critical period in early life. Since most sensory information is transmitted to the cortex *via* the thalamus, it will be of significant interest to understand the precise time window and underlying mechanisms of this critical period in the thalamus. By using *in vitro* whole-cell patch recording in acute brain slices, we found that VPm relay synapses were only sensitive to whisker deprivation from postnatal day 11 (P11) to P14. Whisker deprivation initiated within the P11 to P14 window significantly reduced the amplitude of AMPAR-EPSCs, but not NMDAR-EPSCs when recorded 24 h after whisker removal. From P10 to P11, the timing for entry into the critical period and the kinetics underlying NMDAR-EPSCs function were significantly altered. At P11, NMDAR-EPSCs were less sensitive to ifenprodil, a selective blocker of NR2B-containing NMDAR, and the protein level of NR2A was significantly increased compared to those at P10. At the end of the critical period there were no obvious changes in synaptic properties when compared between P14 and P15. Using calcium imaging, we found that fewer P15 VPm neurons could be excited by the GABAa receptor agonist, muscimol, when compared to P14 VPm neurons; this correlated to an increase in KCC2 expression. Our studies revealed a precise critical period of sensory experience-dependent plasticity in the thalamus featuring distinct molecular mechanisms which occur at the start and end of this critical window.

## Introduction

Synaptic plasticity, the ability of synapses to be strengthened or weakened in response to neuronal activity in the central nervous system, plays a critical role in normal brain function, particularly in terms of learning and memory. In some brain regions, such as the cortex and hippocampus, plasticity can be maintained throughout the entire life (Trachtenberg et al., 2002; Schmidt-Hieber et al., 2004; Neves et al., 2008; Holtmaat and Svoboda, 2009). In contrast, many subcortical regions are thought to exhibit lower levels of plasticity. During development, there are specific time windows, or ‘critical periods,’ when the brain is extremely sensitive to the manipulation of sensory experience. These critical periods, in turn, influence the development of many other sensory aspects and functions within different sensory modalities (Fox, 1992; Zhang et al., 2002; Morishita and Hensch, 2008). Sensory experiences during these critical periods are known to play essential roles in shaping precise neural circuits. Incorrect timing of the beginning and end of these critical periods, or the occurrence of aberrant sensory experiences during these periods, are thought to be significantly related to a range of neurological disorders, including autism and schizophrenia. Therefore, understanding the underlying mechanisms of such influences remains a very important goal.

Previously, extensive studies of sensory experience dependent plasticity have mainly focused on the cortex, usually with specific emphasis upon the visual and somatosensory cortex (Fox and Wong, 2005; Hensch, 2005). Research carried out by Hubel and Wesel on cats, for example, discovered that monocular vision deprivation (MD) during a critical window (from the 4th week after birth to the end of the 3rd month) resulted in a strong shift in cortical responsiveness toward the non-deprived eye and an associated reduction of acuity in the vision of the deprived eye. This phenomenon was named ocular dominance (OD) plasticity (Wiesel and Hubel, 1963; Hubel and Wiesel, 1970). Subsequent studies confirmed that OD plasticity also existed in rodents (Gordon and Stryker, 1996). The maturation of cortical inhibitory networks in the postnatal brain has been proven to be important for initiating the critical period of OD plasticity. However, the precise mechanisms for controlling the ending of this process are not well understood (Fagiolini and Hensch, 2000; Hensch, 2005; Di Cristo et al., 2007; Carulli et al., 2010; Southwell et al., 2010; Davis et al., 2015).

In addition to the cortical area, recent studies have found that subcortical regions, especially the thalamus, may be more plastic in early developmental stages than initially thought. Evidence for this has a similar focus upon both visual and somatosensory systems (Hooks and Chen, 2008; Wang and Zhang, 2008). Synapses formed by the retina and the lateral geniculate nucleus (LGN) undergo extensive elimination and strengthening during early development and are seen to be only sensitive to deprivation of vision experience from P20 to 32 (Hooks and Chen, 2006). In the somatosensory system, tactile information from large whiskers on the rodent snout is relayed to the neocortex primarily through the lemniscal pathway which involves the principal V nucleus (Pr5) in the brainstem, the ventral posterior medial nucleus (VPm) in the thalamus and the paralemniscal pathway (Williams et al., 1994). When whisker sensory experience deprivation was initiated at P13, there was a rapid reduction of AMPAR-EPSCs over a period of 24 h, but not NMDAR-EPSCs at the VPm relay synapse in the thalamus. However, whether there is a critical window for the rapid modification of synaptic properties at the VPm relay synapse which relates to the deprivation of whisker sensory experience remains unclear. Since sensory information is transmitted to the cortex *via* the thalamus, the experience-dependent plasticity in the thalamus, especially during the critical period, must contribute to cortical plasticity. Therefore, understanding the precise time window and underlying mechanisms of this critical period in the thalamus is very important and of significant interest. In this study, we attempted to answer these questions by employing whole-cell patch recording and calcium imaging techniques.

## Results

### The Precise Critical Period for Whisker Sensory Experience-Dependent Plasticity at the VPm Relay Synapse Is from P11 to P14

**FIGURE 1:**
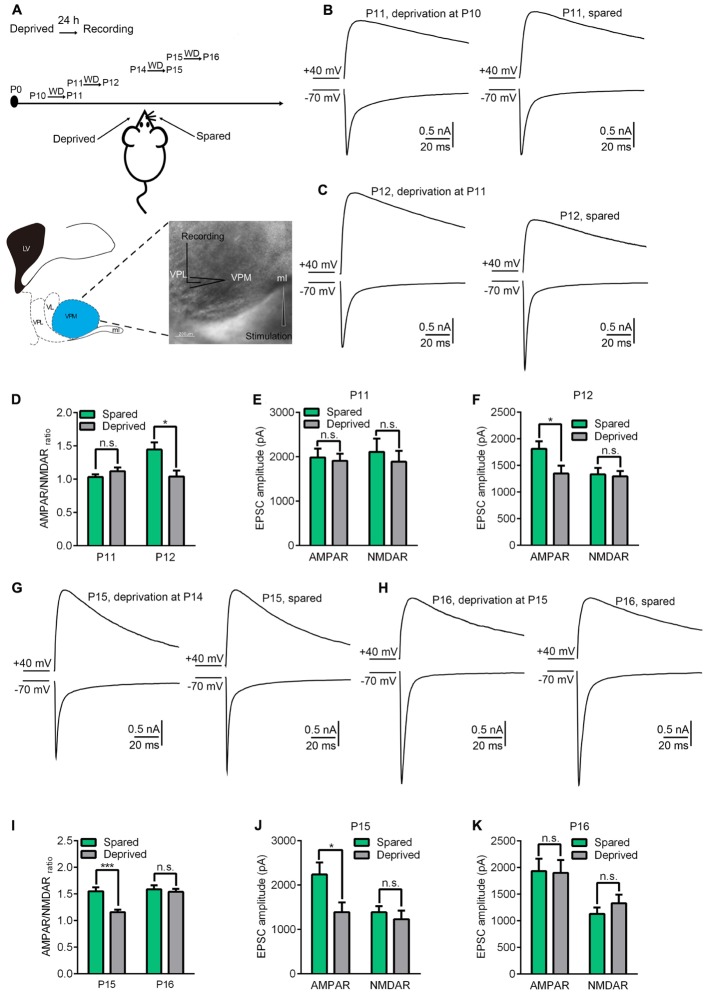
The precise critical window of experience-dependent plasticity in the VPm occurs between P11 and P14. **(A)** Experimental strategy diagram. Whisker deprivation was performed at P10, P11, P14, P15 and the synaptic properties were tested 24 h after each of these times (Upper image). The cartoon (Left) and the image (Right) show the location of the VPm synapse. Whole-cell recordings were acquired at the soma of VPm neurons and a concentric bipolar electrode (FHC) was placed in the medial lemniscus (Lower). Scale bar, 200 μm. **(B,C)** Maximal EPSCs recorded at +40 mV (upper traces) and **–**70 mV (lower traces) in deprived (left traces) or spared (right traces) VPm neurons at P11 **(B)** and P12 **(C)**. **(D)** AMPAR/NMDAR ratio obtained from VPm neurons 24 h after whisker deprivation on P10 or P11. **(E,F)** Peak amplitude of the maximal EPSC-AMPAR and EPSC-NMDAR for deprived and spared neurons on P11 **(E)** and P12 **(F)**. **(G,H)** Maximal EPSCs recorded in deprived (left traces) or spared (right traces) VPm neurons on P15 **(G)** and P16 **(H)**. **(I)** AMPAR/NMDAR ratio obtained from VPm neurons on P15 and P16 1 day after whisker deprivation. **(J,K)** Peak amplitude of maximal EPSC-AMPAR and EPSC-NMDAR for deprived and spared neurons on P15 **(J)** and P16 **(K)**. ^∗^*p* < 0.05, ^∗∗∗^*p* < 0.001, Student’s *t*-test. Error bars indicate SEM.

In a previous study, we found that whisker sensory experience deprivation, beginning at P13, rapidly altered the properties of the VPm relay synapse (Wang and Zhang, 2008). Whisker deprivation caused a significant reduction of AMPAR-EPSCs, but not NMDAR-EPSCs, within 24 h. To investigate the precise time window for whisker sensory experience-dependent plasticity at the VPm, we firstly performed whisker deprivation by plucking out all whiskers gently from one side of the mouse snout at different time-points (P10, P11, P12, P14, and P15). Then, 24 h later, *in vitro* whole cell patch recording was applied to acute brain slices in order to examine the contralateral (deprived) and ipsilateral (spared) neurons in the VPm and to test the effects of whisker deprivation on synaptic properties (**Figure 1A**). Since both the deprived and spared neurons were from the same mouse, results obtained from this preparation were considered to be highly reliable. Maximal AMPAR-EPSCs and NMDAR-EPSCs of the VPm neurons were evoked using the same intensity of stimuli applied to the medial lemniscus when membrane potentials were held at -70 mV and +40 mV, respectively. The AMPAR-mediated component of EPSC was determined by measuring the peak amplitude of EPSC at -70 mV. At +40 mV, AMPAR-EPSC was very small due to a strong inward rectification, and decayed rapidly (Hooks and Chen, 2008; Wang and Zhang, 2008). Thus, the peak amplitude of EPSC at +40 mV was almost entirely mediated by NMDARs. We estimated the NMDAR-mediated component at +40 mV by measuring the amplitude of EPSC at 8 ms after the beginning of EPSC. Our data showed that the AMPAR/NMDAR ratio was altered when whisker deprivation was performed at P11 (**Figures 1C,D**). However, whisker deprivation prior to P11, such as P10, failed to show any change in synaptic properties when examined 24 h later (**Figures 1B,D**). The mean AMPAR/NMDAR ratio recorded at P12 (whisker deprivation was performed at P11) was 1.04 ± 0.33(*n* = 13 cells from 4 mice) for deprived neurons and 1.45 ± 0.44 (*n* = 16 cells from 4 mice) for spared neurons, thus showing a statistically significant difference (p < 0.02). We also found that the observed reduction of AMPAR/NMDAR ratio when recorded at P12 was due to a significant reduction of AMPAR-EPSCs, although NMDAR-EPSCs remained un-affected (**Figure 1F**). In contrast, whisker deprivation at P10 had no such effects on either AMPAR/NMDAR ratio or AMPAR-EPSCs when recorded 24 h later on P11 (**Figures 1D,E**). These results suggested that the start of sensory experience dependent plasticity at the VPm relay synapse occurs on P11.

Next, we used the same strategy to explore the timing of the end of the critical period. We found that whisker deprivation initiated on P15 also failed to alter synaptic properties in deprived neurons as compared to spared neurons when recorded after 24 h (P16) (**Figures 1H,I,K**). However, a significant reduction of the AMPAR/NMDAR ratio in the deprived neurons was observed when whisker deprivation was performed on P14 and recording was conducted on P15 (**Figures 1G,I**) (1.58 ± 0.34 for deprived, *n* = 12 cells from 4 mice; 1.14 ± 0.17 for spared, *n* = 10 cells from 4 mice, *p* < 0.001). Consistent with previous results, the reduction of AMPAR/NMDAR ratio in the deprived neurons was due to a significant reduction of AMPAR-EPSCs (**Figure 1J**). This result suggested that whisker sensory-experience dependent plasticity ends on P15. Collectively, our results indicated that P11 to P14 represents the precise critical window for sensory experience dependent plasticity at the VPm relay synapses.

### The Recruitment of NR2A-Containing NMDARs May Be Responsible for Entry into the Critical Period at the VPm Relay Synapse

**FIGURE 2:**
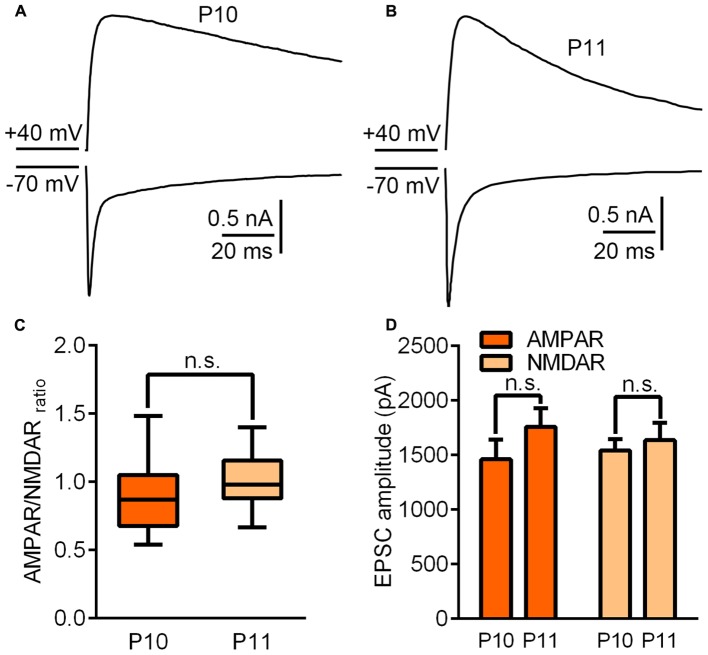
Synaptic properties did not change significantly between P10 and P11. **(A,B)** Sample traces illustrating maximal EPSCs recorded on P10 **(A)** and P11 **(B)**. **(C)** AMPAR/NMDAR ratio recorded on P10 and P11. **(D)** Peak amplitudes of the maximal EPSC-AMPAR and EPSC-NMDAR recorded on P10 and P11. Error bars indicate SEM.

**FIGURE 3:**
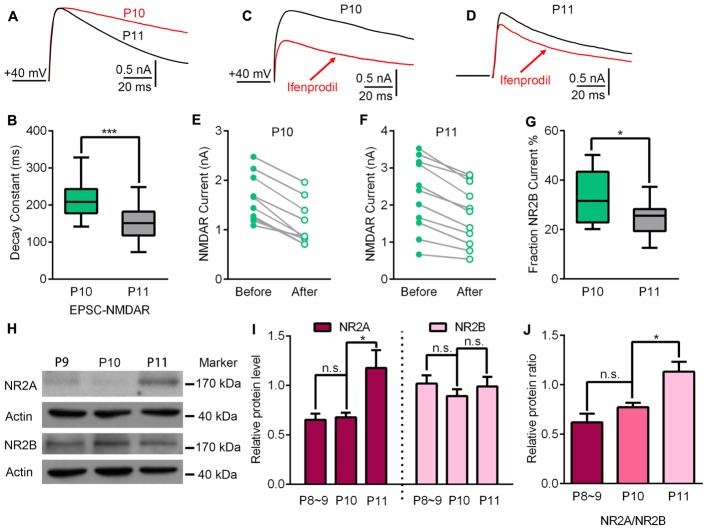
Up-regulation of NR2A-containing NMDA receptors may contribute to the beginning of experience-dependent plasticity at the VPm. **(A)** EPSC-NMDAR recorded on P10 (red) and P11 (black). **(B)** The decay constant of the EPSC-NMDAR current rapidly decreased from P10 to 11 (P10, *n* = 21 cells from 5 mice; P11, *n* = 23 cells from 5 mice). **(C,D)** NMDAR-EPSCs recorded before (black) and after (red) the application of an ifenprodil bath on P10 **(C)** and P11 **(D)**. **(E,F)** Compared to P10 **(E)**, the application of ifenprodil has less effect on the decay constant of NMDAR-EPSCs recorded on P11 **(F)**. **(G)** NR2B-NMDAR-mediated current decreased significantly from P10 to P11. **(H)** Representative data showing quantitative Western blot results using anti-NR2A, anti-NR2B and anti-actin antibodies on P9, P10, and P11. **(I)** The protein levels of NR2A (∼175 kDa), but not NR2B (∼180 kDa), were significantly increased at the beginning of the critical period (P11; P8–9, *n* = 4; P10, *n* = 5; P11, *n* = 4). **(J)** Relative protein level of NR2A/NR2B on P8∼9, P10 and P11. ^∗^*p* < 0.05, ^∗∗∗^*p* < 0.001, Student’s *t*-test. Error bars indicate SEM.

To investigate the underlying mechanisms driving the VPm relay synapse to enter the critical period, we compared VPm synaptic properties between P10 and P11 from C57BL/6J mice without any whisker sensory experience manipulation. We found that neither the AMPAR/NMDAR ratio, nor the maximal AMPAR-EPSCs or NMDAR-EPSCs differed between P10 and P11 (AMPAR/NMDAR ratio: P10 = 0.92 ± 0.27, *n* = 21 cells from 5 mice; P11 = 1.02 ± 0.18, *n* = 23 cells from 5 mice, *p* = 0.14; maximal AMPAR-EPSCs: P10 = 1462 ± 778 pA; P11 = 1760 ± 674 pA, *p* = 0.24; maximal NMDAR-EPSCs: P10 = 1542 ± 458 pA; P11 = 1747 ± 455 pA, *p* = 0.61; **Figure 2**). Interestingly, the kinetic of the NMDAR-EPSCs had significantly changed between P10 and P11. The mean decay constant recorded on P11 was considerably faster than that recorded on P10 (**Figures 3A,B**; *p* < 0.001). It is well accepted that glutamatergic synapses possess a developmental NMDAR-subunit switch from slow decay NR2B-containing synapses to fast decay NR2A-containing synapses, which results in a reduction in the decay constant. Our results indicated that a rapid recruitment of NR2A-containing NMDAR subunits had occurred between P10 and P11. To test this idea, we recorded NMDAR-EPSCs in the presence of a specific antagonist for NR2B-containing NMDARs, ifenprodil. We found that the application of 3 μM ifenprodil dramatically reduced the decay constant of NMDAR-EPSCs on P10 (**Figures 3C,E**), but had less effect on the decay constant when recorded on P11 (**Figures 3D,F**). This result suggested that NMDAR-EPSCs on P10 are more sensitive to ifenprodil than those at P11, indicating fewer NR2B-containing NMDARs on P11 (**Figure 3G**). Next, we used Western blotting to test the change of NMDAR subunits in the VPm at the protein level. Here, we measured the protein levels of NR2A and NR2B subunits from the VPm at different time points, from P8 to P11. Our results showed that levels of the NR2A subunit, and NR2A/NR2B ratio, significantly increased between P10 and P11 (**Figures 3H–J**) although there was no change in levels of the NR2B subunit (**Figures 3H–J**). When comparing levels of the NR2A subunit, NR2B subunit and the NR2A/NR2B ratio, between P8–9 and P10 groups, no significant changes were observed (**Figures 3I,J**). Our results therefore revealed that an increase in the recruitment of NR2A-containing NMDARs in the VPm occurs concurrent with the start of the suggested critical period.

### The Maturation of GABAergic Inhibition Is Concurrent with the Ending of the Critical Period

**FIGURE 4:**
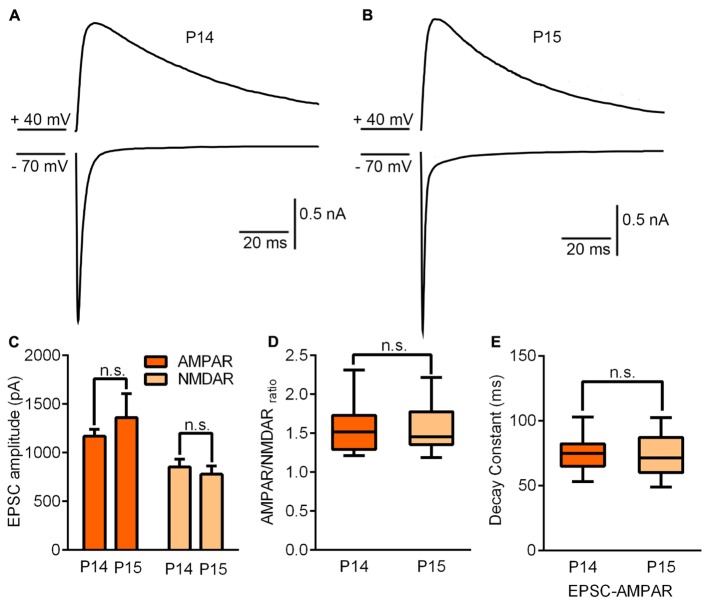
Synaptic properties remained unchanged by the end of the critical period. **(A,B)** Sample traces illustrating maximal EPSCs recorded on P14 **(A)** and P15 **(B)**. **(C)** AMPAR/NMDAR ratio recorded on P14 and P15. **(D)** Peak amplitudes of maximal EPSC-AMPAR and EPSC-NMDAR on P14 and P15 (P14, *n* = 15 cells from 4 mice; P15, *n* = 11 cells from 3 mice). **(E)** The decay constant of the EPSC-NMDAR current did not show any statistically significant difference between P14 and P15. Error bars indicate SEM.

Next, we attempted to determine the possible mechanisms controlling the end of the critical period. To do this, we compared the synaptic properties of B6 mice without any whisker sensory experience manipulation between P14 and P15. No statistically significant differences were observed for AMPAR-EPSCs (*p* = 0.35), NMDAR-EPSCs (*p* = 0.57), AMPAR/NMDAR ratio (*p* = 0.91) or decay constant of NMDAR-EPSCs (*p* = 0.93) (**Figure 4**). Given that the ending of the critical period in the visual cortex was attributed to maturation of GABAergic interneurons, we needed to consider whether there was a correlation between maturation of the GABAergic system and the closure of this critical period in the thalamus.

**FIGURE 5:**
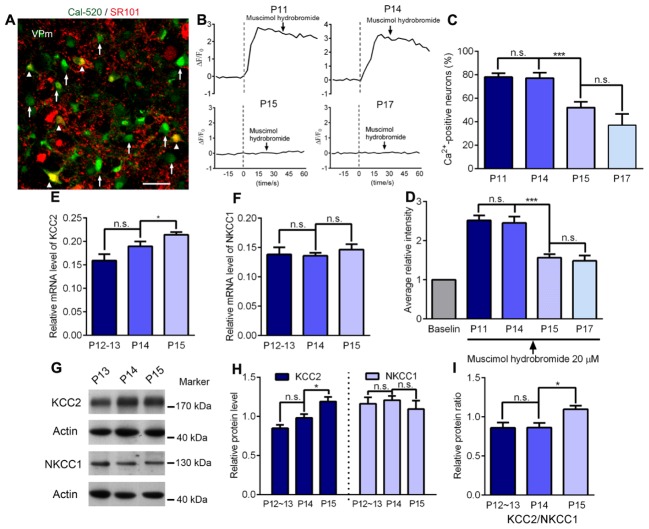
Maturation of GABAergic inhibition is concurrent with the ending of the critical period. **(A)** Confocal image showing VPm cells loaded with the Ca^2+^ indicators Cal, 520 AM (green) and labeled with sulforhodamine 101 (SR101; red). The white arrows indicate VPm neurons while the arrowheads indicate VPm astrocytes. Scale bar, 30 μm. **(B)** Representative [Ca^2+^]_i_ elevation in response to muscimol hydrobromide (20 μM) in VPm neurons on P11, P14, P15 and P17. Arrow indicates muscimol hydrobromide perfusion. **(C,D)** The proportion of Ca^2+^-positive neurons **(C)** and average fluorescence intensity **(D)** decreased sharply at the end of the critical period (P15; P11, *n* = 162 cells; P14, *n* = 83 cells; P15, *n* = 102 cells; P17, *n* = 42 cells). **(E,F)** The mRNA levels of KCC2 **(E)**, but not NKCC1 **(F)**, were significantly increased at the end of the critical period (P12–13, *n* = 4; P14, *n* = 4; P15, *n* = 4). **(G)** Representative data showing quantitative Western blot results using anti-KCC2, anti-NKCC1 and anti-actin antibodies on P13, P14, and P15. **(H)** The protein level of KCC2 (∼175 kDa) was significantly increased at the end of the critical period (P15), but there were no changes in the level of NKCC1 (∼130 kDa; P12–13, *n* = 7; P14, *n* = 5; P15, *n* = 4). **(I)** Relative protein level of KCC2/NKCC1 on P12∼13, P14 and P15. ^∗^*p* < 0.05, ^∗∗∗^*p* < 0.001, Student’s *t*-test. Error bars indicate SEM.

In the new born brain, glutamatergic neurons are excitatory to GABA at first but then change function to become inhibitory at approximately the 2nd week after birth. The time window for this switch is very similar to the end of the critical period for the sensory experience-dependent plasticity at the VPm. Therefore, we wanted to test whether these two events occurred concurrently in the thalamus. Using calcium imaging in acute brain slices, we next investigated the response of VPm neurons incubated in a drug (Cal-520, AM) to the application of 20 μM of the GABAa receptor agonist, muscimol (**Figure 5A**). We found that the proportion of VPm neurons exhibiting an increase in intracellular calcium signaling induced by muscimol hydrobromide was similar when compared between P11 and P14, but was remarkably reduced on P15, and showed a further, less marked, reduction on P17 (**Figures 5B–D**). These results suggest that between P11 and P14, comparable neurons had the capability to be excited by GABA, whereas between P14 and P15, a significantly increased proportion of VPm neurons turn from being excitatory to inhibitory in response to GABA. In line with the calcium imaging results, we found that the mRNA and protein levels of K^+^-Cl^-^ co-transporter 2 (KCC2) that is essential for the transition of excitatory to inhibitory function, had increased remarkably in the VPm between P14 and P15 (**Figures 5E–I**).

## Discussion

By employing electrophysiological recording and calcium imaging techniques, we discovered that a precise and critical time window for sensory experience-dependent plasticity occurs at the VPm relay synapses. Specifically, we made the following exciting discoveries: (1) the precise experience-dependent critical period started from P11 and closed on P14; (2) the recruitment of NR2A-containing NMDARs may be responsible for initiating of this critical window; and (3) the transition from an excitatory to inhibitory response of the VPm neurons to GABA is concurrent with the closure of this critical window.

It is now known that many brain areas have a number of critical periods occurring at various times which are activated and regulated by distinct mechanisms (Hensch, 2004). While a large number of studies have revealed much information relating to the cortex, experience-dependent plasticity in subcortical areas remains poorly understood. In the thalamus, different types of critical periods for sensory experience-dependent plasticity are known to co-exist. The critical period for vision related sensory-experience dependent plasticity at the LGN occurs from P20 to P32, whereas that for sensory experience-dependent plasticity at the VPm relay synapses occurs from P11 to P14. Since mice start to actively explore their environments at around P11–P12, and that their eyes do not open until P13–14, it is reasonable to consider that the critical period of experience-dependent plasticity relating to the somatosensory system occurs somewhat earlier than that of the visual system. Within the somatosensory system, there are also multiple types of critical periods, occurring at different time windows (Erzurumlu and Gaspar, 2012). P3–P4 represents the end of the critical period for structural plasticity following whisker follicle or infraorbital nerve (ION) damage. After P4, whisker follicle or ION damage no longer affects the topographic organization of the barrel structure in the cortex (Van der Loos and Woolsey, 1973). Another type of critical period occurs only prior to P9, where long-term potentiation (LTP) can be induced into the barrel cortex (Lu et al., 2001). These two types of critical period occur earlier than those we found in the thalamus and may possess distinct mechanisms. On the other hand, removal of all but a single whisker (single whisker experience; SWE) can trigger the strengthening of individual glutamatergic synaptic contacts from layer 4 to layer 2/3 neurons, but only during P11 to P13 (Wen and Barth, 2011). This is very similar to the critical period we found in the thalamus. Therefore, the critical periods in the barrel cortex, and in the thalamus, could be mechanistically related. However, sensory experience-dependent plasticity in the barrel cortex involves calcium permeable AMPARs, whereas plasticity in the thalamus is mainly attributed to the GluR3 subunit (Wang et al., 2011). Nevertheless, we cannot exclude the possibility that the initiation and end of these critical periods may share common mechanisms. Total whisker deprivation during the same critical period in the second postnatal week (P12–14) produces profoundly abnormal layer 2/3 receptive fields (Stern et al., 2001). Given that somatosensory information is transmitted to the barrel cortex through the VPm, the onset of active whisking (Welker et al., 1964), the emergence of mature inhibition (Kiser et al., 1998) and weakening of the VPm relay synapse may all occur in anticipation of this period of plasticity.

The glutamatergic synapses of early development stages mostly possess NR2B-containing NMDA receptors which are eventually replaced by NR2A-containing receptors during further development (Monyer et al., 1994; Zhang and Luo, 2013). As NR2A-containing NMDARs exhibit faster decay constants than NR2B-containing NMDARs, a gradual shortening of the NMDAR decay constant was observed between the 1st and 4th week in many brain regions including the cortex and hippocampus (Barth and Malenka, 2001; Gray et al., 2011). In the present study, we found that the NMDAR decay constant reduced dramatically at the VPm relay synapses and was accompanied with a reduction in sensitivity to ifenprodil, but only between P10 and P11. This suggests a significant reduction of NR2B-containing NMDARs on P11 as compared to that on P10. A similar phenomenon was also observed at the retina- superior colliculus synapses (Shi et al., 2000). Additionally, we observed the rapid enhancement of NR2A protein levels at the VPm, indicating that the observed shortening of the NMDAR decay constant may be due to an increase of NR2A-containing NMDARs. NR2A-containing and NR2B-containing NMDARs have been recognized for their differential roles in synapse plasticity, possessing their own distinct kinetics for activation and intracellular signaling pathways (Kim et al., 2005). Studies using culture neurons have shown that NR2B-containing NMDARs play a role in preventing the recruitment of AMPARs, whereas NR2A-containing NMDARs have an opposite role (Hall et al., 2007). Inserting the NR2A subunit into NMDARs could remove the blocking effects produced by NR2B and promote the recruitment of AMPARs. At the VPm relay synapse, AMPARs are known to be mostly composed of GluR3 and GluR4 subunits; the GluR4 subunit is expressed early, followed by the developmental recruitment of GluR3 subunit (Wang et al., 2011). Furthermore, the sensory experience-dependent plasticity at the VPm relay synapse is predominantly attributed to the GluR3 subunit, since GluR4-KO mice exhibit normal experience-dependent plasticity while GluR3-KO mice do not. Thus, the developmental incorporation of NR2A-containing NMDARs, particularly between P10 and P11, may recruit a considerable number of GluR3 subunits into the AMPARs and thus initiate the critical period in the VPm. This idea is consistent with previous studies which found that juvenile mice lacking GluN2A showed reductions of LTP in the superior colliculus and visual cortex (Philpot et al., 2007; Zhao and Constantine-Paton, 2007), and were deficient in visual experience-dependent synaptic plasticity (Philpot et al., 2007).

Studies of the visual cortex indicate that the excitatory-inhibitory balance may lead to a structural consolidation that eventually terminates the critical period (Hensch, 2004), although the mechanisms underlying this process still remain unclear. There is evidence for several possible mechanisms: persistently potent inhibition, neuromodulatory desensitization and an increase in structural factors that inhibit neurite remodeling (Espinosa and Stryker, 2012). One interesting feature of glutamatergic cells in the CNS is that they are excitatory to GABA during early development and then switch to being inhibitory at around the 2nd week after birth. This is due to the early expression of the Na^+^-K^+^-Cl^-^ co-transporter (NKCC1) and the developmental increase in expression of KCC2, resulting in distinct intracellular Cl^-^ concentrations at different ages (Li and Xu, 2008; He et al., 2014). Recent research using the NKCC1 inhibitor bumetanide, prolonged the critical period of plasticity in the visual cortex and suggested a link between KNCC1 and closure of the critical period (Deidda et al., 2015). As glutamatergic neurons are found in other brain regions, the VPm neurons are also excited by GABA during early life, as indicated by the fact that the majority of VPm neurons younger than P14 exhibit a transient increase of calcium signaling in response to muscimol. Interestingly, we observed a rapid reduction in the proportion e of neurons which were activated by muscimol on P15, when compared to those on P14. In line with calcium imaging results, our electrophysiological recordings also indicate a switch from excitation to inhibition of the VPm neurons to muscimol stimulation (data not shown). This was possibly caused by the rapid increase of KCC2 levels between P14 and P15. It is important to note that our results only provide a temporal correlation between the increased levels of KCC2 and the end of experience-dependent plasticity. Indeed, it is unlikely that the switch of excitation to inhibition of the VPm neurons to GABA is the cause of the critical period closure, because this switch occurred gradually and heterogeneously, while almost all VPm neurons exit the critical period at the same time after P14.

## Materials and Methods

### Animals

All of our experiments involved C57BL/6J (B6) mice, aged between P10 (postnatal 10 days with the day of birth considered to be P0) and P17. All experimental procedures were conducted under the Guidelines of Zhejiang University Animal Experimentation Committee. The protocol was approved by the Zhejiang University animal experimentation committee.

### Whisker Deprivation

C57BL/6J (B6) mice aged between P10 and P15 were anesthetized by isoflurane. All large whiskers were gently pulled out on one side of the snout with a pair of forceps, without incurring damage to the follicles.

### Slice Preparation

Sagittal slices were obtained using methods described previously (Yang et al., 2016). In brief, mice were anesthetized with sodium pentobarbital and then perfused with ice-cold oxygenated slicing solution. After decapitation, brain were removed rapidly for sectioning in ice-cold slicing solution containing 110 mM choline chloride, 7 mM MgCl_2_⋅6H_2_O, 2.5 mM KCl, 0.5 mM CaCl_2_⋅H_2_O, 1.3 mM NaH_2_PO_4_, 25 mM NaHCO_3_, 20 mM glucose, saturated with 95% O_2_ and 5% CO_2_. For all VPm recordings, 300 μm slices were prepared using a vibratome (Leica VT1000). Slices were recovered for 1 h at physiological temperature and then transferred to a recording chamber for recording in artificial cerebrospinal fluid (ACSF) containing 125 mM NaCl, 2.5 mM KCl, 2 mM CaCl_2_⋅H_2_O, 1.3 mM MgCl_2_⋅6H_2_O, 1.3 mM NaH_2_PO_4_, 25 mM NaHCO_3_, 10 mM glucose.

### Patch-Clamp Recording

Recordings were undertaken at room temperature. The pipette solution contained 110 mM caesium methylsulfate, 20 mM TEA-Cl, 15 mM CsCl, 4 mM ATP-Mg, 0.3 mM GTP, 0.5 mM EGTA, 10 mM Hepes, 4.0 mM QX-314 and 1.0 mM spermine (pH 7.2; adjusted to 270–280 mmol kg-1 with sucrose). Electrodes had resistances between 2 and 4 MΩ. Whole-cell voltage-clamp recordings were acquired from the soma of VPm neurons with an Axopatch 200B amplifier and Digidata 1322A with pCLAMP 8.1 software (Molecular Devices). Signals were filtered at 2 kHz and digitized at 10 kHz. The series resistance (Rs) was < 20 MΩ with no compensation. A concentric bipolar electrode (World Precision Instruments) was placed in the medial lemniscus, and stimuli (100 μs, 0.01–1.0 mA) were applied at 0.1 Hz. GABAergic transmission was blocked by 100 μM picrotoxin in the bath.

### Western Blot (WB)

Mice aged between P9 and P15 were used, and brain slices were prepared as described previously. Then, VPm subfield tissue was carefully dissected out with scalpel blades. Tissues were then dissolved with 250 μl of lysis buffer containing RIPA strong (Beyotime, P0013B, China), PMSF (Beyotime, ST505, China), phosphatase inhibitor cocktail 2 (Sigma, 5726) and PhosSTOP Phosphatase Inhibitor Cocktail (Roche, 4906845001). The lysate was pipetted several times to ensure all solid parts were washed down. To ensure efficient lysis, samples were mixed on a rotary shaker for 15 min at 4°C before centrifugation at 12,000 *g* for 20 min at 4°C. The supernatant was collected and protein concentration determined using a BCA protein assay kit (Thermo Fisher Scientific). Each sample was uniformly diluted to 1 μg/μl for WB.

Western blot samples were added to 4xSDS sample buffers before loading and were subsequently boiled for 5 min. 20 μl of sample was loaded to each well. After electrophoresis, proteins were transferred to nitrocellulose membranes (Whatman, GE Healthcare) and incubated in blocking buffer containing 5% BSA dissolved in TBST for 1 h at room temperature. Membranes were then incubated overnight with primary antibodies at 4°C. After washing three times with TBST, the blots were incubated with horseradish peroxidase-conjugated secondary antibodies for 1 h at room temperature. The blots were then detected on X-ray film with a chemiluminescent substrate (Thermofisher Scientific, 34080) after washing four times with TBST. Immunoreactivity of the bands was then quantified by densitometric analysis using Quantity One software (Bio-Rad). The relative amounts of NR2A, NR2B, NKCC1 and KCC2 proteins were quantified by normalizing the optical density of the correct molecular weight band to that of actin on the same gel.

The following antibodies were used in the Western blotting experiment: GluN2B C-terminal antibody (mice monoclonal antibody produced in-house, WB 1:800), GluN2A antibody (Abcam ab133265, 1:1000), NKCC1 antibody (C-14; SantaCruz, sc-21547, 1:300), KCC2 antibody (Upstate, 07-432, 1:500), anti-β-actin antibody (A5316, Sigma, 1:10000). The secondary antibodies were goat anti-rabbit conjugated IgG-HRP and goat anti-mouse conjugated IgG-HRP (31420, 31460, Pierce, 1:10,000) and donkey anti-goat IgG HRP (Abcam, ab97110, 1:20,000).

### Quantitative Real-Time PCR

Brain slices were prepared as described previously. Then, VPm subfield tissue was carefully dissected out with scalpel blades. Total RNA was extracted from brain tissue using Trizol (TaKaRa) and reversely transcribed into cDNA with the PrimeScript RT Reagent Kit (TaKaRa) according to the manufacturer’s instructions. qRT-PCR was then carried out using an CFX96 Real-Time PCR Detection System (Bio-Rad). Gene expression levels were calculated according to the 1/Δct method and the relative amounts of mRNA were normalized to β-actin as an internal control. Primer sequences were searched defined by the NCBI Gene ID in PrimerBank^1^. Validated primers were selected as follows: forward primer for KCC2 5′-GGGCAGAGAGTACGATGGC-3′; reverse primer for KCC2 5′-TGGGGTAGGTTGGTGTAGTTG-3′ (amplicon size 111 bp); forward primer for NKCC1 5′-TTCCGCGTGAACTTCGTGG-3′; reverse primer for NKCC1 5′-TTGGTGTGGGTGTCATAGTAGT-3′ (amplicon size 197 bp); forward primer for β-actin 5′-AACAGTCCGCCTAGAAGCAC-3′; reverse primer for β-actin 5′-CGTTGACATCCGTAAAGACC-3′ (amplicon size 281 bp).

### Brain Slice Ca^2+^ Imaging

For Ca^2+^ imaging in the VPm, we used C57 mice aged between P11 and P17; acute brain slices were prepared as described previously. Then, sagittal slices containing VPm were incubated with the Ca^2+^ indicator, Cal-520, AM (AATBioquest) and sulforhodamine 101 (SR101) for 1 h at room temperature in oxygenated ACSF. Cal-520 is a non-selective Ca^2+^ indicator which can simultaneously label both neurons and glial cells, whereas SR101 specifically labels glial cells. During imaging, VPm cells loaded with Cal-520, AM, but not SR101, were confirmed to be neurons. Images were acquired every 3 s using an Olympus FV-1200 confocal microscope. The average fluorescence intensity was measured from analysis boxes placed over the cell-bodies of neurons. When the baseline was stable, we added 20 μM muscimol hydrobromide (Sigma, M1523) to activate GABA_A_-R in the VPm neurons. Increases in fluorescence intensity over baseline were then calculated for each trace and are reported herein as ΔF/F0.

### Statistical Analysis

All statistical analyses were performed using Prism (GraphPad Prism software). Differences between the two groups were assessed using a *t*-test. Data are presented as mean ± standard error of the mean (SEM). Differences were considered to be statistically significant when *p* < 0.05.

## Author Contributions

LP, JY, and HW designed the project, and LP and JY performed electrophysiology, western blot and calcium imaging experiments, and collected and analyzed the data. QY, YL, and XW helped to collect the data. LZ, HL, CX, YS, and HW interpreted the results and commented on the manuscript. HW wrote the manuscript and supervised all aspects of the project.

## Conflict of Interest Statement

The authors declare that the research was conducted in the absence of any commercial or financial relationships that could be construed as a potential conflict of interest.

## Footnotes

1 https://pga.mgh.harvard.edu/primerbank/
